# Commentary: Essential elements that contribute to the recovery of persons with severe mental illness: a systematic scoping study

**DOI:** 10.3389/fpsyt.2025.1563684

**Published:** 2025-07-21

**Authors:** Hideki Muramatsu

**Affiliations:** Graduate School of Arts and Sciences, The Open University of Japan, Chiba, Japan

**Keywords:** recovery, severe mental illness, expert-by-experience, social construction, subjective well-being

## 1 Introduction

The systematic scoping review of Jaiswal et al. (1) identified three pillars of the recovery journey: relationship, sense of meaning, and participation. Their discussion of research limitations highlighted the need for greater client involvement in critical research and emphasized the influence of environmental factors, particularly for individuals with severe mental illness. This commentary offers a novel socially constructed perspective (2) on the subjective wellbeing of personal recovery for persons with severe mental illness based on an expert-by-experience (EBE) approach (3) that integrates lived experience and academic insight. Knowledge of recovery is informed by the personal and subjective experiences of those who have lived with mental illness (4–6). This paper highlights the contextual experience of individuals with severe mental illness, expanding the reader’s responsiveness profile—a comprehensive list of factors to which an individual is responsive (7).

## 2 “Don’t force us to recover too quickly”

The concept of recovery-oriented care is gaining international recognition (8), and the relationship between individuals with mental illness and service providers may influence recovery (1, 9). The utility of the pragmatic approach to recovery—comprising the three pillars of relationship, sense of meaning, and participation (1)—is evaluated from my perspective using an EBE approach and with a history of significant mental difficulties (3). My lived experience includes chronic crises with severe depression, frequent panic attacks, ego derangement, and cognitive dysfunction (10). In 2024, during a breakout session of a joint meeting of mental health professionals, persons with severe mental illness, and their families in Japan (https://kanto24th.jnpf.net/), a person with mental illness said, “Do not force us to recover.” I have facilitated over 80 self-help group sessions, where I have heard similar statements echoed by participants, and comparable statements reported internationally (11). This discussion draws attention to recovery aspects that professionals often overlook (7).

## 3 Sense of purpose for recovery

“Sense of meaning” suggests that creating a sense of purpose is crucial for recovery and is closely linked to hope and subjective wellbeing (1, 12, 13). When targeting persons with severe mental illness, the demand for clear results can negatively influence recipients’ subjective wellbeing. When I was severely mentally ill (3), my father insisted that I adopt a sense of purpose, despite my lack of readiness. I was compelled to engage in positive thinking when I felt hopeless, which caused intense anguish and highlighted a stark contrast with the acceptance and empathy necessary for recovery (1). My father and my individual subjectivities interacted to construct my intersubjective negative subjectivity. In addition, individuals may remain in stagnant phases of recovery or experience regressions toward illness/struggle, making improvements in subjective wellbeing difficult to achieve (6, 14). Valued subjective experiences, such as wellbeing, are crucial to the recovery process; therefore, a key clinical goal is to promote wellbeing. Emphasizing wellbeing over deficits during assessment is a central feature of person-centered planning (15). However, expectations that individuals in early recovery should achieve positive emotions or rapid progress can be counterproductive. Such expectations defy the core principles of person-centered care. Moreover, efforts to accelerate social reintegration through social structures may, in some cases, harm the subjectivity of individuals with severe mental illness in a socially constructed manner.

## 4 Intersubjective wellbeing

When I lacked a clear sense of purpose and had not yet observed measurable clinical gains, the supportive presence of others sustained my subjective wellbeing. My personal experience indicates that social relationships (16)—particularly reciprocal relationships with informal caregivers (17) and family relationships (1, 9, 16)—are a critical environmental resource. This example represents only a partial element of social structure. Although society encompasses various domains (e.g., family, school, workplace, social institutions of the state, and cultural norms), this paper focuses on family (society) as a micro-community. The CHIME framework for personal recovery in mental health comprises five key components: connectedness, hope and optimism about the future, identity, meaning in life, and empowerment (18). While this framework includes connectedness, its individualistic, Cartesian dualistic foundation tends to underestimate the relational dimensions of recovery (16).

Accordingly, in this article, I argue that subjective wellbeing is not an individualistic perspective of the Cartesian dualistic worldview’s individualistic concept (16). Subjective wellbeing includes intersubjective wellbeing constructed through relationships with others, which is a key element of socially constructed recovery (2). Intersubjective wellbeing is not merely an individual concept of subjectivity, but rather an intersubjective concept based on mutuality. Psychological knowledge emerges through intersubjective exchanges that develop over time rather than exclusively from the “outside-in” objectivity or the “inside-out” subjectivity (19). Intersubjectivity refers to the capacity to share and to coordinate experiences with others in everyday social interactions (20).

For example, in episodes of acute illness during recovery, when I could not articulate a sense of purpose, I engaged in regressive play with my mother—activities without a clear goal, such as watching animation, singing, and dancing. My subjective happiness was incorporated into my exhausted hope, becoming an intersubjective happiness through my interactions with her. This example highlights that hope and recovery involve influences from others, which are incorporated into oneself and become a part of recovery (2). The mutual care and reciprocal empowerment embedded in this relationship facilitated my recovery (10). From my EBE perspective, when a sufficient sense of subjective wellbeing is difficult to maintain, the influence of intersubjectivity—a socially constructed understanding that recognizes the influence of others’ subjectivity—must be considered. This example centers on micro-level social structural relationships; however, if you belong to a medical institution, you may be subject to cultural influences (such as the biomedical or recovery models) in an intersubjective manner.

## 5 Discussion

Individuals with severe mental illness may say, “Don’t force us to recover too quickly.” External pressure from professionals or family members to recover when readiness is low can cause significant distress (11). My lived experience as an EBE and that of my peers confirm that insufficient readiness for recovery breeds hopelessness and impedes subjective wellbeing. In these situations, the environment plays a crucial role (1). Social hierarchies, stigma, and other pressures and structures may be internalized by people with severe mental illnesses, with detrimental effects (2); thus, it is necessary to consider various social influences ranging from micro to macro levels that influence subjective wellbeing. This perspective introduces a social model of thinking that contrasts with the individualistic responsibility emphasized in the CHIME framework and underscores the need for person-centered care (2). By fostering relationships as an intersubjective element, professionals can help replenish the depleted subjective wellbeing of individuals (21, 22). The person-centered care model has been mandated in the US in long-term care settings for over 30 years and is recognized as an effective approach for people with complex needs (23). In addition, there is broad consensus that person-centered care is a core value in general practice (24). Furthermore, as humans are social beings, relationships with others are fundamental to person-centered care.

Centering on the family as a micro unit of society, this paper shows how the attitudes of experts, family, and peers may influence an individual’s subjectivity, and elements such as “human warmth and hope” may be interactively incorporated into individuals in a socially constructed manner. Discussions within this context are significant because they promote a socially constructive (2) and supportive perspective while expanding the responsiveness profiles of individuals (7). Research on recovery must avoid being biased toward an individualistic perspective and rather consider the influence of social factors when attributing the causes of subjective wellbeing. I hope that the concepts of intersubjectivity and social construction (2) will prove useful when considering the role of the environment in recovery (1).
